# The Effects of CAMPATH-1H on Cell Viability Do Not Correlate to the CD52 Density on the Cell Surface

**DOI:** 10.1371/journal.pone.0103254

**Published:** 2014-07-22

**Authors:** Fuiyee Lee, Martha Luevano, Paul Veys, Kwee Yong, Alejandro Madrigal, Bronwen E. Shaw, Aurore Saudemont

**Affiliations:** 1 University College London, Cancer Institute, London, United Kingdom; 2 Anthony Nolan Research Institute, London, United Kingdom; 3 Great Ormond Street Hospital for Children NHS, London, United Kingdom; 4 Royal Marsden NHS Foundation Trust, Sutton, United Kingdom; CNRS, France

## Abstract

Graft versus host disease (GvHD) is one of the main complications after hematological stem cell transplantation (HSCT). CAMPATH-1H is used in the pre-transplant conditioning regimen to effectively reduce GvHD by targeting CD52 antigens on T cells resulting in their depletion. Information regarding CD52 expression and the effects of CAMPATH-1H on immune cells is scant and limited to peripheral blood (PB) T and B cells. To date, the effects of CAMPATH-1H on cord blood (CB) cells has not been studied. Here we aimed to analyze CD52 expression and the effects of CAMPATH-1H on fresh or frozen, resting or activated, PB mononuclear cells (PBMC) and CB mononuclear cells (CBMC). In resting state, CD52 expression was higher in CB than PB T cell subsets (653.66±26.68 vs 453.32±19.2) and B cells (622.2±20.65 vs 612.0±9.101) except for natural killer (NK) cells where CD52 levels were higher in PB (421.0±9.857) than CB (334.3±9.559). In contrast, CD52 levels were comparable across all cell types after activation. CAMPATH-1H depleted resting cells more effectively than activated cells with approximately 80–95% of apoptosis observed with low levels of necrosis. There was no direct correlation between cell surface CD52 density and depleting effects of CAMPATH-1H. In addition, no difference in cell viability was noted when different concentrations of CAMPATH-1H were used. CD52 was not expressed on HSC but began to be expressed as the cells differentiate, implying that CAMPATH-1H could potentially affect HSC differentiation and proliferation. Our study provides insightful information, which contributes to the better understanding in the use of CAMPATH-1H as part of the conditioning regime in HSCT.

## Introduction

Hematopoietic stem cell transplantation (HSCT) is currently used to treat hematological and non-hematological malignancies. However, graft versus host disease (GvHD) remains one of the main drawbacks after HSCT [1]. CAMPATH-1H, also known as Alemtuzumab, is an engineered IgG1κ monoclonal antibody (MoAb) generated from a murine Fab segment conjugated to a human Fc fragment [2], which depletes cells by targeting CD52 antigens on the surface of T cells via antibody dependent cell cytotoxicity (ADCC) [3], complement dependent cytotoxicity (CDC) [4], [5], and induction of apoptosis [6]. CD52 is expressed on lymphocytes, monocytes, eosinophils, and macrophages [7], [8], [9]. Clinical data has shown that CAMPATH-1H is an efficient means to achieve rapid T cell depletion in patients undergoing allogeneic HSCT [10], [11]. The incorporation of CAMPATH-1H into the conditioning regimen as GvHD prophylaxis lowers the incidence of GvHD in patients after HSCT [12], [13], [14], [15]. The use of CAMPATH-1H is common when bone marrow (BM) or mobilized peripheral blood (PB) are used as a source of hematopoietic stem cells (HSC) but is currently not routinely used in cord blood transplantation (CBT).

Data regarding the levels of CD52 expression on the cell surface is mainly limited to PB T cells and B cells whereas CD52 expression on cord blood (CB) cells has not been determined. It has been reported that CD52 expression was the highest in PB B cells, with memory B cells expressing higher CD52 levels than naïve B cells [16], whereas CD52 levels were lower in PB T cells [17]. Among all lymphocytes, natural killer (NK) cells exhibited the lowest level of CD52 expression [17], [18]. It is currently not known whether regulatory T (Treg) cells and natural killer T (NKT) cells expressed CD52. However, it is of interest as these cells play important roles in reducing the risk of GvHD while maintaining graft versus leukemia (GvL) effects [19], [20], highlighting the need for a more detailed study covering a broader range of immune cell types. It still remains unclear whether hematopoietic stem cells (HSC) express CD52 antigens [7], [8], [21], [22], [23], [24]. HSC generate all lymphoid and myeloid cells, which all express CD52, however it is unknown when CD52 starts being expressed and what the impact of CAMPATH-1H on the differentiation of HSC is.

It has been hypothesized that there is a direct correlation between the density of CD52 antigens on immune cells and the efficacy of CAMPATH-1H in depleting those cells. One study reported that CD52 expression levels conferred differences in sensitivity towards CAMPATH-1H [17]. Notably, it has been shown that the cytolytic effect of CAMPATH-1H was greater in B and T cells with high CD52 density but NK cells that had lower CD52 levels were not depleted as efficiently [17].

It is crucial to study whether CAMPATH-1H affects CB cells in a similar manner as PB cells to determine whether CAMPATH-1H could also be used as part of the conditioning prophylaxis for CBT. Unlike mPB or BM transplantation (BMT), CBT is carried out using readily available frozen CB units and both CB and PB consist of a mixture of resting and activated cells. Therefore, the aim of this study was to investigate the qualitative expression of CD52 on resting or activated, fresh or frozen, PBMC or CBMC. We also analyzed the effects of CAMPATH-1H on the viability of the aforementioned cell types from both sources to investigate whether there is a correlation between the sensitivity towards CAMPATH-1H and the qualitative levels of CD52 on immune cells. Moreover, using an *in vitro* model of differentiation of HSC into NK cells, CD52 expression and the impact of CAMPATH-1H on HSC and on the differentiating cells was assessed.

## Materials and Methods

### Peripheral blood and cord blood samples

All CB samples were obtained with prior consent and ethical committee approval from the Anthony Nolan Cord Blood bank (Research Ethics Committee reference 10/H0405/27). Fully informed written consent was obtained from pregnant mothers. PB samples were obtained from healthy volunteers under written informed consent. The study had full ethical approval from the Anthony Nolan and Royal Free Hospital Research Ethics Committee. Frozen PBCD34^+^ samples were provided by Prof Kwee Yong, University College London Hospitals (UCLH) using chemotherapy/G-CSF. Informed written consent, with a protocol approved by the UCL/UCLH Committee on the Ethics of Human Research, was obtained. CB samples were processed within 24 hours upon collection.

### Cell isolation and cryopreservation

Mononuclear cells were isolated from CB or heparinized PB by density-gradient centrifugation using Ficoll-Paque PLUS (GE Healthcare Bio-sciences, Uppsala, Sweden) or lympholyte-H (VWR, Leicestershire, UK) respectively. CB CD34+ cells were isolated using the CD34 MicroBead kit (Miltenyi Biotec, Surrey, UK) according to a published protocol [25]. Cord blood mononuclear cells (CBMC) and peripheral blood mononuclear cells (PBMC) were frozen down in 10% dimethylsuphoxide (Sigma, Poole, UK) and 90% fetal bovine serum (FBS) (Lonza, Verviers, Belgium) at a concentration of 5×10^6^ cells/ml and were kept at −80°C for 24 hours followed by another 24 hours in liquid nitrogen.

### Cell culture

Resting cells were treated with five different concentrations (0.05 µg/ml; 0.1 µg/ml; 0.5 µg/ml; 1.0 µg/ml; 1.2 µg/ml) of CAMPATH-1H. For activated cells, T cells and Treg cells were activated for 48 hours using 100 and 1000 IU/ml of IL-2 (Prospec, Israel) respectively and the T cell activation/expansion kit (MACS, Miltenyi Biotec, Surrey, UK) as recommended by the manufacturer, however, a 1∶1 loaded Anti-Biotin MACSiBead Particle to cell ratio was used to achieve optimal activation. NK cells from CBMC and PBMC were activated in the presence of 1000 or 200 IU/ml of IL-2 respectively for six days at 37°C as previously described [26]. CAMPATH-1H (Genzyme Corporation, Oxford, UK), 30 µg/ml, was a kind gift from Dr P Veys, Great Ormond Street Hospital, London, UK. As suggested by Li *et al*, human serum was used as a source of complement and was added to the cells at a final concentration of 10% (v/v) [27]. Activated cells were treated with two different concentrations (0.1 µg/ml and 1.2 µg/ml) of CAMPATH-1H. Various cell types were characterized as follows: naïve CD4 and CD8 T cells (CD3+CD4+CD45RA+ and CD3+CD4−CD45RA+ respectively), memory CD4 and CD8 T cells (CD3+CD4+CD45RO+ and CD3+CD4−CD45RO+ respectively); NK cells (CD56+CD3–); NKT cells (CD56+CD3+); B cells (CD19+); Treg cells (CD4+CD25+FoxP3+ or CD4+CD25^high^CD127^low^) (**Figure S1**). Activated cells were characterized as follows: activated CD4 and CD8 T cells (CD3+CD4+CD69+CD25+ and CD3+CD4−CD69+CD25+); activated Treg cells (CD4+FoxP3+CTLA-4+GITR+); activated NK cells (CD56+CD3−CD69+NKp44+) (**Figure S2**). Apoptotic cells were gated as Annexin V+ and necrotic cells were gated as 7AAD+.

### Flow cytometry analysis

The following antibodies were purchased from Becton and Dickinson (Oxford, UK): 7-AAD, Annexin V, anti-CD3 (SK7), anti-CD4 (RPA-t4. SK3), anti-CD7 (124-1D1), anti-CD19 (4G7), anti-CD25 (2A3), anti-CD33 (WM53), anti-CD34 (581), anti-CD45 (HI30), anti-CD45RA (HI186), anti-CD45RO (UCHL1), anti-CD56 (B159), anti-CD69 (L78), anti-CD127 (hIL-7R-M21), and CTLA-4 (BNI3). Additional antibodies such as anti-CD52 (HI186) and NKp44 (P44.8) were purchased from Cambridge Bioscience (Cambridge, UK) whereas anti-CD19 (4G7 2ES) and GITR (110416) were purchased from R&D Systems (Abingdon, UK). Intracellular staining for FoxP3 (PCH101) was carried out with the FoxP3/Transcription factor staining buffer set (eBioscience, Hatfield, UK) according to manufacturer recommendations. All samples were acquired using CellQuest software version 3.3 and a FACS Calibur flow cytometer (Becton and Dickinson, Oxford, UK). Data were analyzed using FlowJo software (TreeStar, USA).

### HSC differentiation into NK cells

EL08.1D2 cells were cultured as previously described [28]. HSC were plated over 2000 irradiated EL08.1D2 cells in a medium consisting of 2∶1 (vol:vol) mix of Dulbecco’s Modification of Eagle’s Medium (DMEM) with 4.5 g/L glucose, L-glutamine 1640, and sodium pyruvate/Ham’s F12 Medium (all from Lonza, Verviers, Belgium) supplemented with 50 µM beta mercaptoethanol (β-ME), 50 µM ethanolamine, 20 mg/L ascorbic acid, 50 µg/L sodium selenite, 1% penicillin/streptomycin and 20% heat-inactivated human AB serum (all from Sigma, Poole, UK). Different interleukins such as 10 ng/ml IL-15, 5 ng/ml IL-3 (only for the first week; R&D System, Abingdon, UK), 20 ng/ml IL-7, 20 ng/ml c-kit ligand stem cell factor (SCF) and 10 ng/ml Flt3 ligand were added up to 21 days of culture and only 10 ng/ml IL-15 were added from day 21 to day 35 (all from Prospec, Israel). Cultures underwent weekly hemi-depletion. Lymphoid progenitors were characterized as CD45+CD7+ whereas myeloid progenitors as CD45+CD33+.

### Colony Forming Unit assay

CFU assays were performed to investigate if CAMPATH affects the ability of HSC in forming colonies *in vitro*. CB or mPB CD34+ cells were resuspended in Iscove’s Modified Dulbecco’s Medium (IMDM) with 2% FBS and seeded in MethoCult (all from Stem Cell Technologies, Grenoble, France). HSC were treated with two different concentrations (0.1 µg/ml and 1.2 µg/ml) of CAMPATH-1H and numbers of colonies were counted 14 days after seeding of HSC.

### Statistics

Statistical analyses were performed using the non-parametric unpaired *t* test, where p-value<0.05 were considered statistically significant, in GraphPad Prism software (GraphPad, San Diego, CA). Taken multiple comparisons into account, Bonferroni adjustment of four was applied to analyze the p-value for each cell type. Any results described as statistically significant had a Bonferroni-adjusted p-value of <0.05 after adjusting for the number of conditions tested for each cell type from both CB and PB. All data were presented as mean ± SD.

## Results

### Activated CB lymphocytes exhibit lower expression of CD52

**Figure 1:**
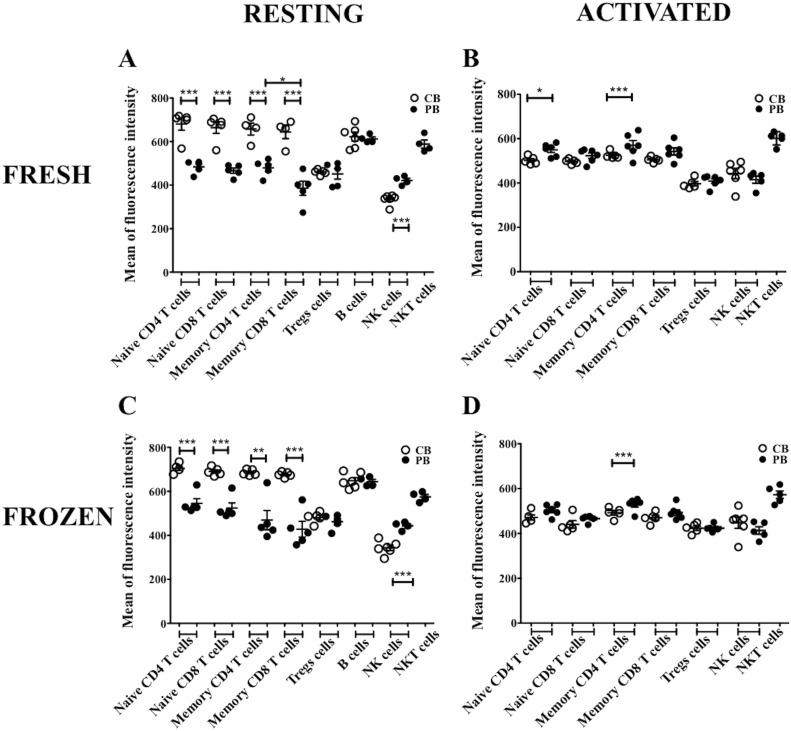
CD52 expression on different CB and PB cell subsets. **A and C** show the level of CD52 expression in fresh and frozen resting immune cells respectively. **B and D** show CD52 expression in fresh and frozen activated immune cells respectively. *represents p value<0.05, **represents p value<0.005, ***represents p value<0.005 (n = 5).

CD52 expression was evaluated on various PB and CB cell types by measuring CD52 mean fluorescence intensity (MFI) by flow cytometry. In resting state, naïve and memory T cells from freshly isolated CBMC expressed significantly higher CD52 levels than PBMC (p<0.05). However, comparable CD52 levels were observed in CB and PB Treg cells (459.4±5.437 and 451.2±23.89 respectively) and B cells (612.9±9.101 and 622.2±20.65 respectively). Higher CD52 levels in PB NK cells than CB NK cells were found (421.01±9.857 *vs* 334.3±9.559) (p<0.0005) (**Figure 1A**). In freshly isolated CBMC, CD52 expression was the highest in naïve and memory T cell subsets and in B cells (680.8±28.35–622.2±20.65). Treg cells expressed relatively lower levels of CD52 levels (459.4±5.437, p<0.0005) than T and B cells while NK cells exhibited the lowest CD52 expression (334.3±9.559, p<0.0005) in freshly isolated CBMC. However, in freshly isolated PBMC, CD52 expression was higher in B cells and NKT cells (612.0±9.101 and 588.5±18.61 respectively) than T cell subsets and NK cells (484.2±12.99 and 421.0±9.857 respectively, p<0.0005) (**Figure 1A**). NKT cells were only studied in PBMC due to the scarcity of this cell type in CB.

In contrast, compared to resting CBMC, activated CBMC expressed lower levels of CD52 except for NK cells, which exhibited higher CD52 levels in activated state (442.5±29.43 activated *vs* 334.3±9.559 resting, p<0.005) (**Figure 1B**). Naïve and memory T cells from activated PBMC expressed higher CD52 levels as compared to T cells from resting PBMC (546.22±15.34 activated *vs* 484.2±12.99 resting, p<0.05) while CD52 expression on Treg cells, NK cells and NKT cells was similar in resting and activated PBMC (**Figure 1B**). Levels of CD52 in activated PBMC and CBMC were identical across all cell types except for naïve CD4 T cells (549.3±12.33 PB *vs* 502.0±8.044 CB, p<0.05) and memory CD4 T cells (570.2±21.30 PB *vs* 524.6±7.033 CB, p<0.05) for which it was higher in PB than CB (**Figure 1B**). Notably, CD52 expression was similar in all cell subsets in frozen and freshly CBMC and PBMC, whether the cells were resting or after activation (**figure 1C and D**). Overall, no difference in CD52 expression was observed between naïve and memory CD4 and CD8 T cell subsets in all conditions tested except for memory CD4 T cells in resting PBMC that expressed significantly higher CD52 levels than their CD8 T cell counterpart (p<0.05) (**Figure 1A–D**). Phenotypic characterization of the aforementioned cell types and activated cells are shown in **Figure S1 and S2** respectively.

### CBMC and PBMC are more resistant to CAMPATH killing after activation

We next investigated to what extent CD52 expression levels affect CAMPATH-1H’s depleting activity. The viability of various cell types was analyzed at different time points after treatment with CAMPATH-1H using 7-AAD and Annexin V. Apoptotic cells were identified as 7-AAD- Annexin V+ while necrotic cells were identified as double positive. Although some differences were noted in the viability of resting and activated cells 24 hours after treatment with different concentrations of CAMPATH-1H, these differences were not statistically significant (**Figure S3**). Therefore, thereafter, only two concentrations of CAMPATH-1H (0.1 µg/ml and 1.2 µg/ml) were used.

**Figure 2:**
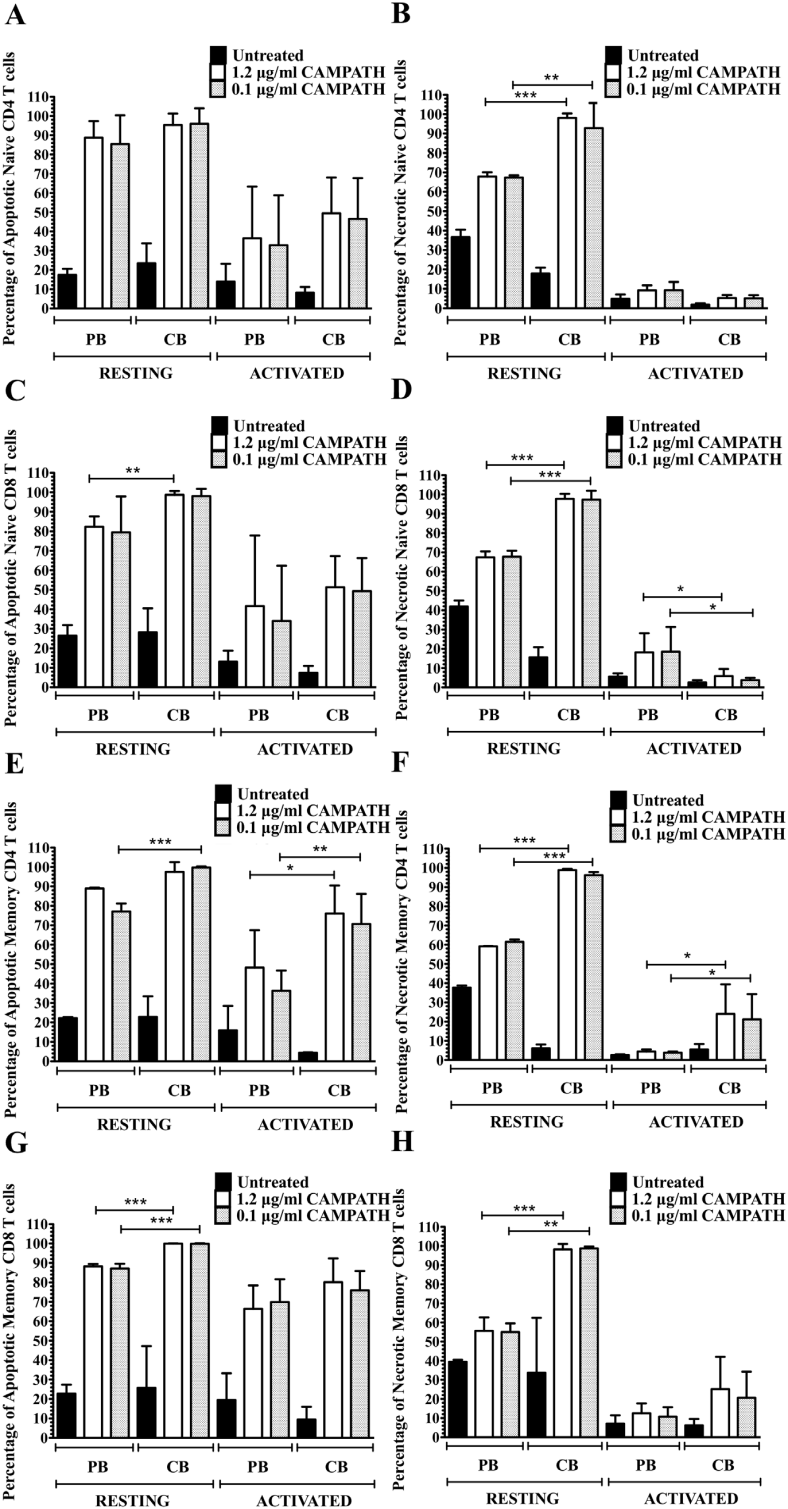
Viability of PB and CB naïve and memory T cells after treatment with CAMPATH. **A, C, E, and G** show the percentage of apoptotic cells in resting/activated naïve CD4, naïve CD8, memory CD4, and memory CD8 T cells respectively. **B, D, F, and H** show the percentage of necrosis in resting/activated naïve CD4, naïve CD8, memory CD4, and memory CD8 T cells respectively. *represents p value<0.05, **represents p value<0.005, ***represents p value<0.005. Significant differences were observed between resting and activated naïve and memory T cells across all fresh samples with p value<0.005 (n = 5).

**Figure 3:**
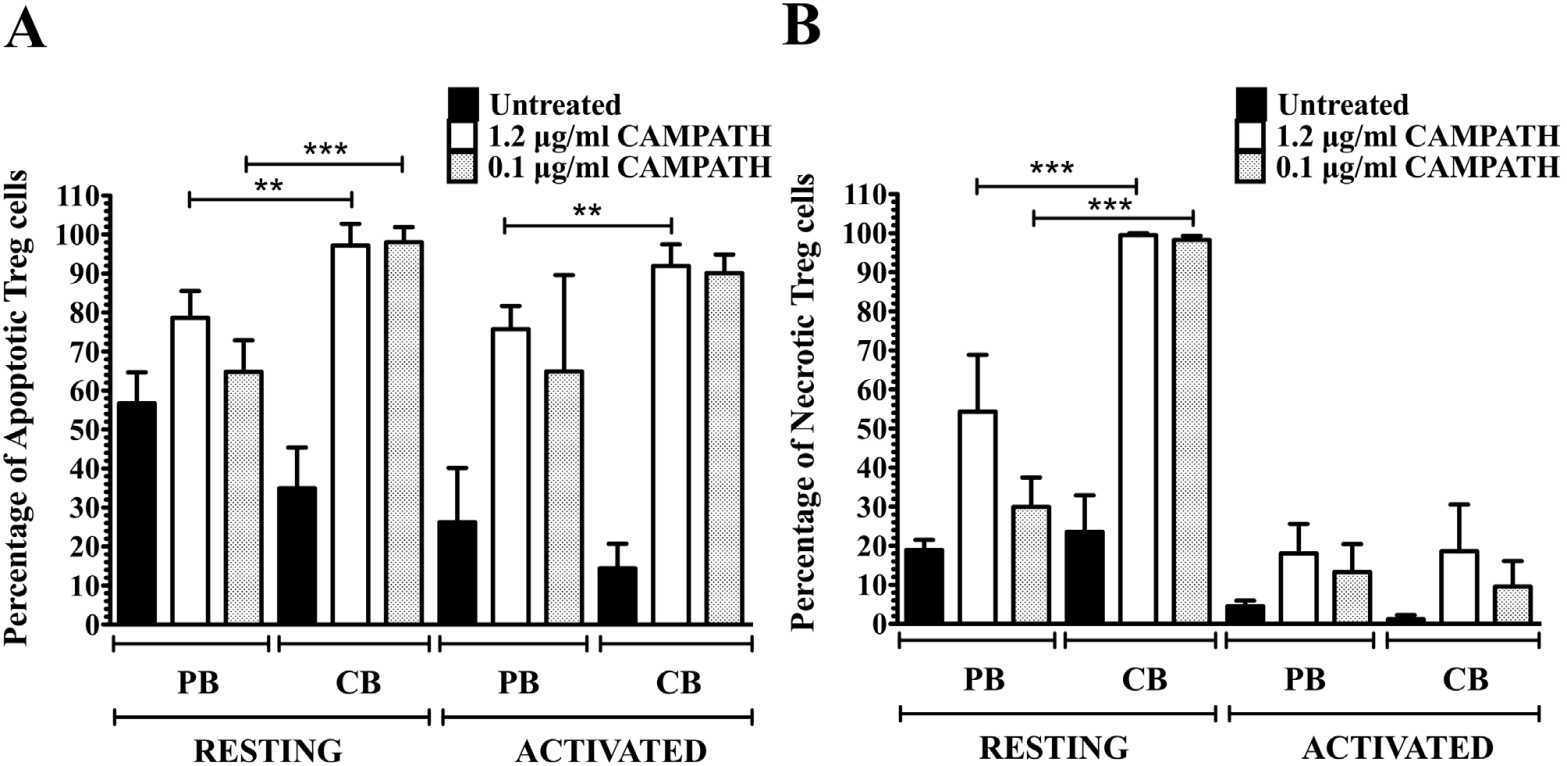
Viability of PB and CB Treg cells after treatment with CAMPATH. **A** shows the percentage of apoptotic cells in resting/activated Treg cells. **B** shows the percentage of necrosis in resting/activated Treg cells. *represents p value<0.05, **represents p value<0.002, ***represents p value<0.005. Significant differences were observed between resting and activated Treg cells across all fresh samples with p value<0.005 (n = 5).

**Figure 4:**
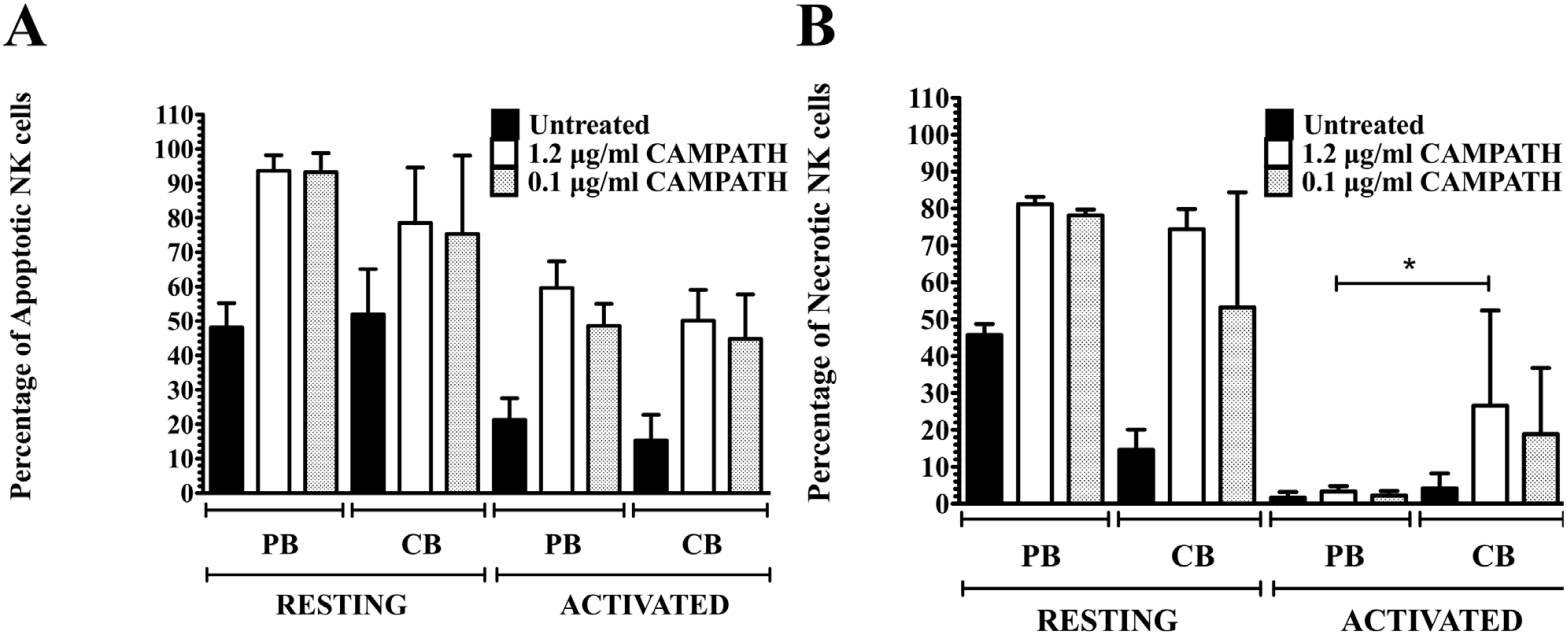
Viability of PB and CB NK cells after treatment with CAMPATH. **A** shows the percentage of apoptosis in resting/activated NK cells. **B** shows the percentage of necrosis in resting/activated NK cells. *represents p value<0.05. Significant differences were observed between resting and activated NK cells across all fresh samples with p value<0.005 (n = 5).

The depleting effect of CAMPATH-1H was significantly greater in resting CB than PB cells with almost 100% and 70% necrotic cells for naïve CD4 T cell subsets respectively. In contrast, activated naïve CD4 T cells from both CB and PB were more resistant to CAMPATH-1H with significantly less apoptosis and minimal necrosis observed (p<0.05) (**Figure 2A and B**). Higher percentages of cell death were observed in resting naïve CD8 T cells and activated memory CD4 and CD8 T cells (p<0.05) (**Figure 2C–H**). Higher level of apoptosis was noted after treatment with CAMPATH-1H in memory CD4 and CD8 T cells from CB and PB (**Figure 2E and G**) than in naïve T cell subsets (p<0.05) (**Figure 2A and C**). Activated Treg cells were more resistant to CAMPATH-1H with less than 20% of necrotic cells observed as compared to resting Treg cells (95% necrotic Treg cells in CB and 30–55% necrotic Treg cells in PB, p<0.0005) (**Figure 3A and B**). Percentages of apoptotic Treg cells did not differ between resting and activated cells in both CB and PB (**Figure 3A**) as well as in naïve and memory T cells from frozen PBMC and CBMC (**Figure S4–S6**). Interestingly, the impact of CAMPATH-1H was greater in CB T cell subsets with higher percentages of apoptosis and necrosis noted than in PB T cell subsets (p<0.05). However, the percentages of apoptotic and necrotic NK cells did not differ significantly between CB and PB after treatment with CAMPATH-1H (**Figure 4A and B**). Low percentage of necrotic NK cells were observed in activated PB and CB (<5% and <30% respectively) whereas 50–80% of NK cells were necrotic in resting PB and CB, p<0.0003 (**Figure 4B**). Moreover, the effects of CAMPATH-1H on NKT cells were similar to T cells with 70–80% of necrosis in resting CB and PB NKT cells and activated NKT cells being more resistant to the drug. The same trend was observed in both fresh and frozen NKT cells (**Figure S7A–D**). The effects of CAMPATH-1H on B cells were investigated only in resting CB and PB cells. There were 70–95% of apoptosis and approximately 70% of necrosis noted in, fresh and frozen, CB and PB B cells (**Figure S8A and B**).

**Figure 5:**
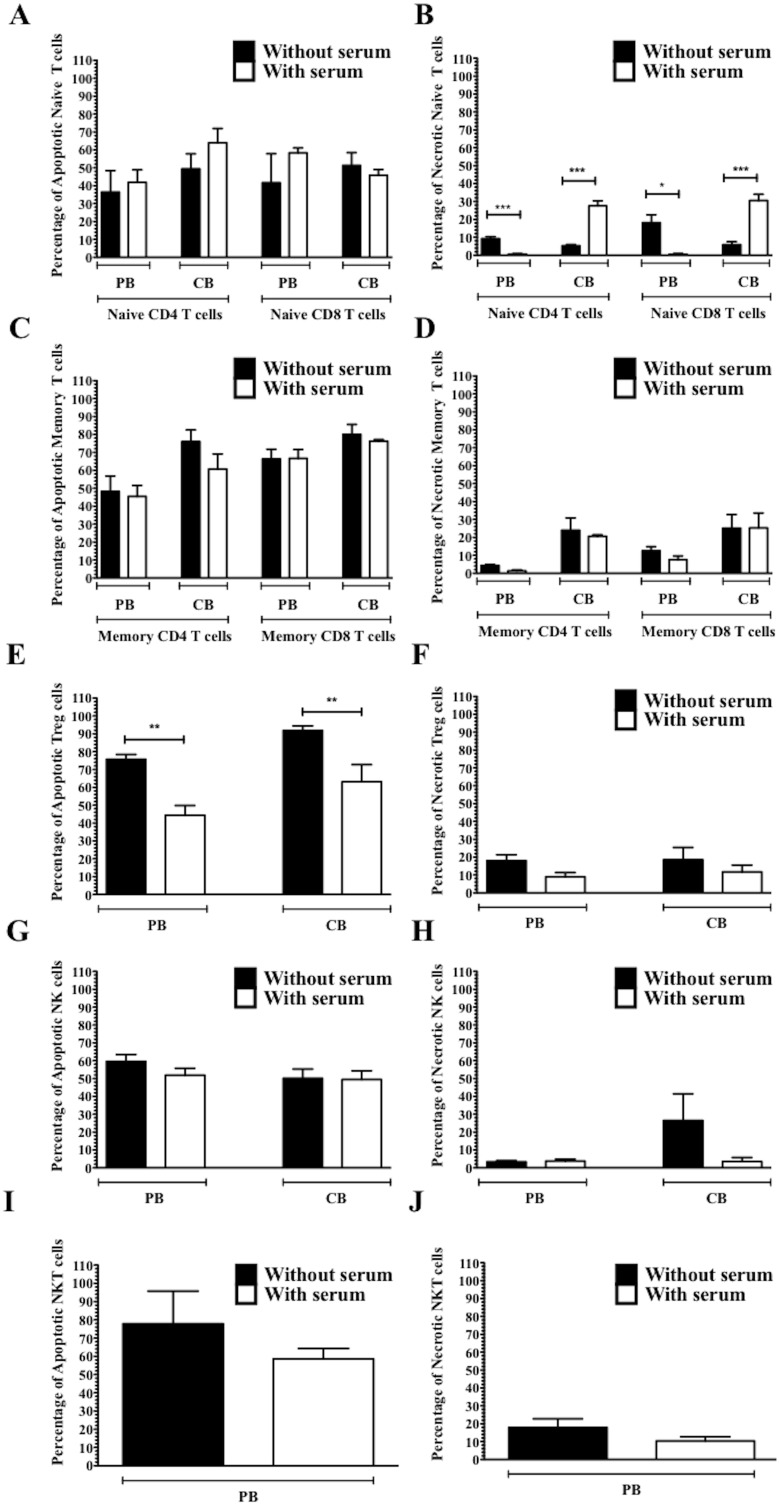
Viability of activated PB and CB immune cells after treatment with CAMPATH-1H in the presence or absence of complement. **A, C, E, G, and I** show the percentage of apoptotic cells in naïve T cells, memory T cells, Treg cells, NK cells, and NKT cells respectively. **B, D, F, H, and J** show the percentage of necrosis in naïve T cells, memory T cells, Treg cells, NK cells, and NKT cells respectively. *represents p value<0.05, **represents p value<0.005, ***represents p value<0.005 (n = 4/5).

In addition, the presence of serum as a source of complement did not alter the impact of CAMPATH-1H on the viability of the cells (**Figure 5 A–J**) except for activated CB and PB naïve CD4 and CD8 T cells, and Treg cells. There was approximately 23% more necrotic naïve T cells in CB, significantly less necrotic naïve T cells in PB when serum was present, p<0.05 (**Figure 5B**) while there was significantly less apoptosis in both CB and PB Treg cells when serum was present, p<0.005 (**Figure 5 E**).

### Hematopoietic progenitor cells, not HSC, express CD52

**Figure 6:**
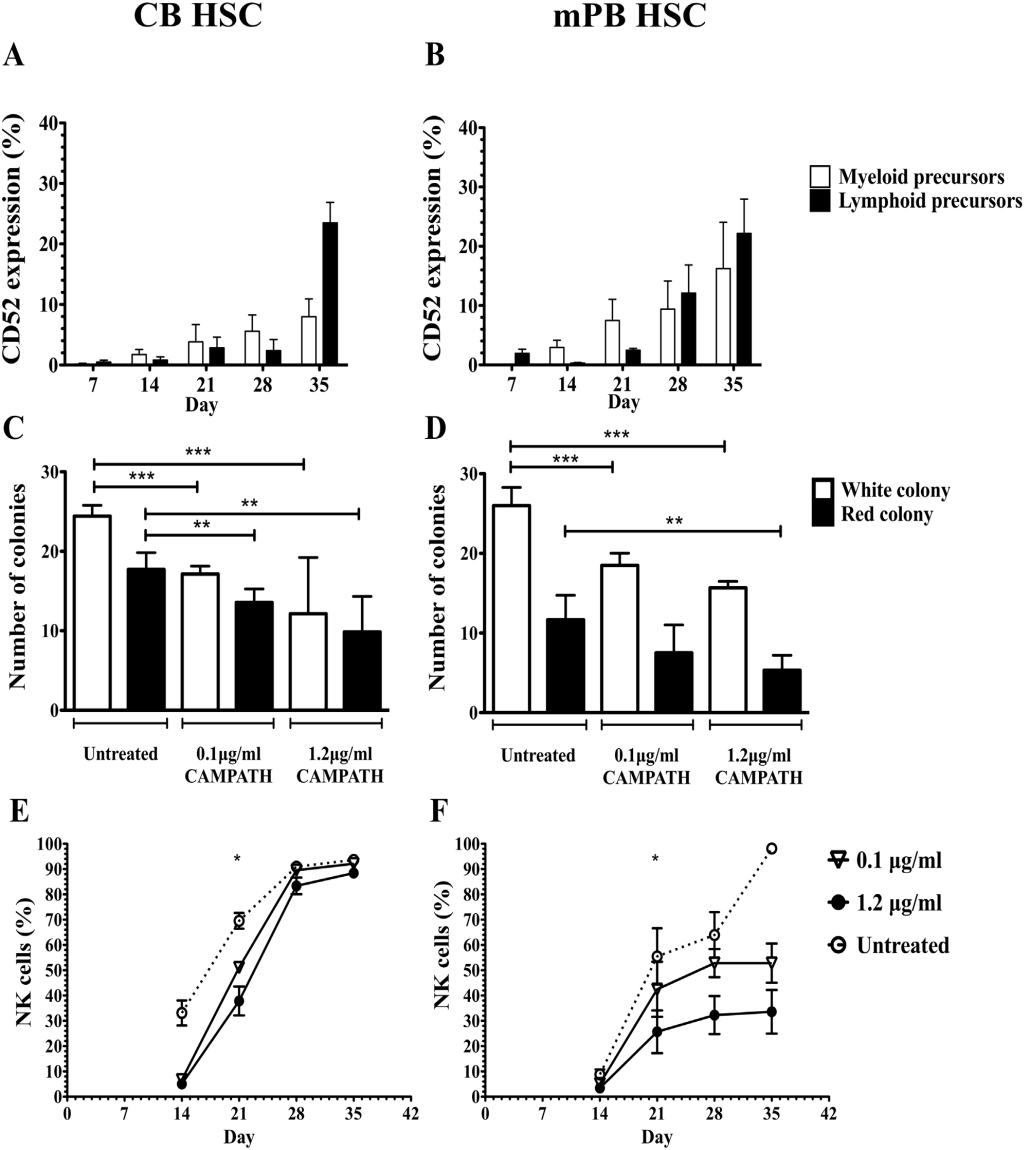
CD52 expression and effects of CAMPATH on HSC and HSPC. **A and B** show the level of CD52 expression in CD45^high/low^ lymphoid and myeloid progenitors in cultures of CB and PB HSC respectively. **C and D** show the number of colony from CB SC and mPB SC respectively at day 14. **E and F** show the percentage of NK cells formed during the 35 days of culture of CB SC and mPB SC respectively. *represents p value<0.05, **represents p value<0.005, ***represents p value<0.005 (n = 3).

HSC generate all blood lineages and contradicting data has been reported on whether HSC express CD52 or not. Therefore, we examined CD52 expression on HSC and hematopoietic progenitors by using an *in vitro* model of HSC differentiation into NK cells [28]. Studies showed that NK cells could be generated from both lymphoid and myeloid lineages [28], [29]; therefore, CD52 expression was investigated on both types of progenitors. Characterization of the lymphoid and myeloid progenitors is shown in **Figure S9**. We found that CD52 was not expressed in CB and mPB HSC (**Figure 6A and B**). However, CD52 expression was detectable from day 21 onwards as NK cells were generated from lymphoid and myeloid progenitors (**Figure 6A and B**). This data suggests that even though CD52 is not expressed on HSC, CD52 begins to be expressed as they differentiate.

HSC proliferate and differentiate into different types of colonies *in vitro*. A significant reduction in the number of white and red colonies formed by CAMPATH-1H treated HSC as compared to untreated HSC for CB (**Figure 6C**) and mPB HSC (**Figure 6D**) was observed (p<0.005).

To test whether CAMPATH-1H affects HSC differentiation into NK cells, HSC were treated with two concentrations (0.1 µg/ml and 1.2 µg/ml) of CAMPATH-1H at days 0 and 7 of HSC differentiation to attempt to approximate the long half-life of CAMPATH-1H present in patients after HSCT. The generation of NK cells from treated CB HSC was reduced at days 14 and 21 as compared to untreated cells (p<0.03). NK cell generation from CB HSC was affected significantly with lower percentage of NK cells in a dose dependent manner at day 21 (p<0.05) (**Figure 6E**). However, no difference was observed between the percentage of NK cells generated from CAMPATH-1H treated or untreated CB HSC at days 28 and 35 of culture. The effects of CAMPATH-1H were more apparent in mobilized PB (mPB) progenitors as significant differences in NK cell generation from CAMPATH-1H treated and untreated mPB HSC were noted in a dose dependent manner at day 21 (p<0.05). However, the percentage of NK cells generated from CAMPATH-1H treated PB HSC plateaued from day 28 onwards while percentage of NK cells from untreated PB HSC increased drastically at days 28 and 35.

## Discussion

It has been speculated that the CD52 density on the cell surface and the cytolytic effects of CAMPATH-1H are directly correlated [17]. Even though we found that CD52 expression levels where the highest in T cells, B cells, and NKT cells and the lowest in NK cells as reported in other studies [17], [18], [30], we observed that the depleting effect of CAMPATH-1H was comparable across all cell types in resting state. However, activated cells were more resistant to CAMPATH-1H effects with minimal necrosis noted. When serum was added as a source of complement to the assay, the depleting effects of CAMPATH-1H were similar except for activated CB naïve CD4 and CD8 T cells where 30% necrosis was observed. Thus, unlike data from Rao *et al* where CD52 density and cytolytic effects of CAMPATH-1H are closely related [17], our data indicate that the level of CD52 expression on the cell surface may not be a good indicator of the depleting effect of the drug. Elter *et al* also mentioned that the correlation between the level of CD52 expression and lymphocyte elimination rate of CAMPATH-1H is still to be justified by clinical data, as there are other factors such as HSC source, age, and timing of CAMPATH-1H administration that could potentially contribute to the clinical outcome in patients [31].

In addition, the current study shows that the depleting effects of CAMPATH-1H was similar between fresh and frozen cells, which postulates that freezing and thawing of CB units in clinics should not alter the clinical outcome. Besides, no difference was noted in the viability of CBMC and PBMC after treatment with different concentrations of CAMPATH-1H, which suggests that low dose of CAMPATH-1H may be sufficient to effectively reduce GvHD without compromising immune recovery or increasing risk of infection in transplanted patients. Several studies reported that 10–20 mg total dose is sufficient to achieve similar outcome as those treated with total dose of 100 mg of CAMPATH-1H while improving *in vivo* clearance of the drug, thus leading to better immune reconstitution [32], [33], [34], [35].

Some studies reported that HSC do not express CD52 [7], [8] while others suggest they do [22], [23], [24]. In our study, CD52 antigens were not detected on HSC but CD52 began to be expressed as hematopoietic progenitors differentiate into myeloid or lymphoid cells. CAMPATH-1H has a long half-life *in vivo* and can still be detected in the serum of patients up to 30 days after HSCT [33], [36], which potentially delay immune reconstitution in patients resulting in increased risk of viral infections [14], [37], [38], [39]. Our data showed significant differences in the formation of colonies between treated and untreated CB and PB progenitors implying that CAMPATH-1H interfered with proliferation and differentiation of HSC. However, Gilleece *et al* suggested that CD34+ HSC express CD52, however no difference in the number of colonies formed between treated and untreated BM hematopoietic progenitors was observed [22]. This might due to the high number of CD34 enriched cells seeded (10^5^ or 5×10^3^ cells per ml) compared to 10^3^ in 3 ml HSC seeded in our study. Interestingly, a study published by Lim *et al* mentioned that CAMPATH-1H significantly enhanced *ex vivo* expansion of CB CD34+ cells and total nucleated cells with data showing 1.31±0.04 folds increase in total CFU expansion [40]. In contrast to our study where only 10^3^ HSC were used in the absence of human complement, Lim et al seeded cells obtained from day 0 and day 14 of the *ex vivo* expansion cultures of CB CD34+ cells [40] leading to the differences in the outcome of the CFU assays.

Using a HSC differentiation model, we demonstrated that CAMPATH-1H impacts on the generation of NK cells up to day 21 of culture where influence of different concentrations of CAMPATH-1H was minimal on CB progenitors but more profound on mPB progenitors. Penack *et al* reported that, even with a very low serum concentration of CAMPATH-1H (<1 µg/ml), NK cells were still very sensitive towards the remaining CAMPATH-1H in the system with >30% necrosis detected and recovery of functional NK cells were dramatically affected [38]. Functionality study on the NK cells generated from the HSC culture could be done to investigate if they are capable of retaining GvL effects.

In summary, no direct correlation between cell surface CD52 density and the depleting effects of CAMPATH-1H on CB and PB immune cells was noted. The comparable findings that the lowest and the highest concentration of CAMPATH-1H used in this study suggest that further reduction in the total dose of CAMPATH-1H in conditioning regimen may provide adequate GvHD prophylaxis without hampering the process of immune reconstitution by facilitating the clearance of the drug *in vivo* after HSCT. Although CAMPATH-1H is not the preferred GvHD prophylactic agent in CBT setting, recently there were two studies that reported durable engraftment in patients treated with low doses of CAMPATH-1H, however high doses of the drug hampered immune recovery [41], [42]. The current study also demonstrated that CB immune cells were equally affected by CAMPATH-1H as PB immune cells even when the lowest dose of CAMPATH-1H was used. Therefore, unless an optimum dosing schedule is established for CBT, the incorporation of CAMPATH-1H in the conditioning regimen pre-HSCT will further delay the restoration of immune cells as seen in conditioning with other T cell depleting antibody such as ATG [43]. Taken together, our study provides a better understanding of the effects of CAMPATH on various immune cell types in both CB and PB, which could be translated clinically to achieve maximal CAMPATH-1H efficacy without delaying immune reconstitution.

## Supporting Information

Figure S1 Phenotypic characterization of T cell subsets, NK cells, NKT cells, and B cells. **A (i)** Naïve T cells were characterized as CD3+CD45RA+, R1. **A (ii)** From R1, naïve CD4 T cells, R2, and naïve CD8 T cells, R3, were identified. **B (i)** Memory T cells were characterized as CD3+CD45RA+, R4. **B (ii)** From R4, memory CD4 T cells, R5, and memory CD8 T cells, R6, were identified. Treg cells were characterized by two methods: **C (i) and (ii)** CD4+CD25+ cells were gated in R7 and, from R7, Foxp3+ cells were gated; **D (i) and (ii)** CD4+ cells were gated in R9 and CD25+CD127low cells, R10, were subsequently gated from R9. **E** NKT cells were characterized as CD56+CD3+, R11, while NK cells were identified as CD56+CD3–. **F** B cells were identified as CD19+, R13.
(TIFF)

Figure S2 Characterization of activated T cells, Treg cells, and NK cells. **A (i)** T cells were identified as CD4 T cells and CD8 T cells by CD3+CD4+, R1, and CD3+CD4−, R2, respectively. **A (ii)** Activated T cells were gated as CD25+CD69+ cells, R3. **B (i)** CD4+ cells were characterized in R4. **B (ii)** From R4, the activated Treg cells were gated as FoxP3+CTLA-4+, R5. **B (iii)** Activated Treg cells were also positive for GITR staining, R6. **C (i)** NK cells were characterized as CD56+CD3–, R7. **C (ii)** From R7, activated NK cells were identified as CD69+NKp44+.
(TIFF)

Figure S3 Percentages of live fresh/frozen PB/CB CD4 T cells after treatment with CAMPATH. Five concentrations ranging from 0.05 µg/ml to 1.2 µg/ml were used to treat the cells and compared with untreated cells at 24 hours after CAMPATH treatment. No significant difference was observed among the 5 different concentrations of CAMPATH used.
(TIFF)

Figure S4 Viability of frozen naïve and memory PB and CB T cells after treatment with CAMPATH. **A, C, E, and G** show the percentage of apoptotic cells in resting or activated naïve CD4, naïve CD8, memory CD4, and memory CD8 T cells respectively. **B, D, F, and H** show the percentage of necrosis in resting/activated naïve CD4, naïve CD8, memory CD4, and memory CD8 T cells respectively. *represents p value<0.05, **represents p value<0.002 (n = 5).
(TIFF)

Figure S5 Viability of frozen PB and CB Treg cells after treatment with CAMPATH. **A** shows the percentage of apoptotic cells in resting or activated Treg cells. **B** shows the percentage of necrosis in resting/activated Treg cells. *represents p value<0.05, **represents p value<0.002, ***represents p value<0.001 (n = 5).
(TIFF)

Figure S6 Viability of frozen PB and CB NK cells after treatment with CAMPATH. **A** shows the percentage of apoptosis in resting/activated NK cells. **B** shows the percentage of necrosis in resting or activated NK cells. *represents p value<0.05 (n = 5).
(TIFF)

Figure S7 Viability of fresh and frozen PB NKT cells after treatment with CAMPATH. **A and C** show the percentage of apoptosis in resting/activated NKT cells. **B and D** show the percentage of necrosis in resting or activated NKT cells. ***represents p value<0.001 (n = 5).
(TIFF)

Figure S8 Viability of resting PB and CB B cells. **A** shows the percentage of apoptosis in fresh/frozen B cells. **B** shows the percentage of necrosis in fresh/frozen B cells.
(TIFF)

Figure S9 Characterization of lymphoid and myeloid progenitors. **A (i)** CD45^low^CD7+ and CD45^high^CD7+ CB lymphoid progenitors were gated in R1 and R2 respectively. **A (ii)** CB myeloid progenitors were identified as CD45^low^CD33+ and CD45^high^CD33+ in R3 and R4 respectively. **B (i)** PB lymphoid progenitors were gated as CD45+CD7+, R5. **B (ii)** Myeloid progenitors derived from PB were identified as CD45+CD33+, R6.
(TIFF)
