# Supplementary figures and images for: The Effects of CAMPATH-1H on Cell Viability Do Not Correlate to the CD52 Density on the Cell Surface
Source: PLoS One. 2014 Jul 22;9(7):e103254. doi: 10.1371/journal.pone.0103254 (PMC4106894; doi:10.1371/journal.pone.0103254)

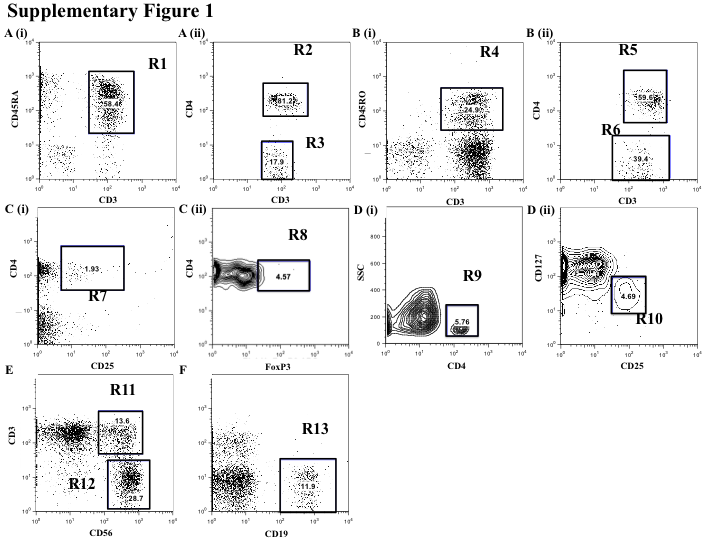

Supplement: Figure S1 — Phenotypic characterization of T cell subsets, NK cells, NKT cells, and B cells. A (i) Naïve T cells were characterized as CD3+CD45RA+, R1. A (ii) From R1, naïve CD4 T cells, R2, and naïve CD8 T cells, R3, were identified. B (i) Memory T cells were characterized as CD3+CD45RA+, R4. B (ii) From R4, memory CD4 T cells, R5, and memory CD8 T cells, R6, were identified. Treg cells were characterized by two methods: C (i) and (ii) CD4+CD25+ cells were gated in R7 and, from R7, Foxp3+ cells were gated; D (i) and (ii) CD4+ cells were gated in R9 and CD25+CD127low cells, R10, were subsequently gated from R9. E NKT cells were characterized as CD56+CD3+, R11, while NK cells were identified as CD56+CD3–. F B cells were identified as CD19+, R13. (TIFF) [file pone.0103254.s001.tiff]

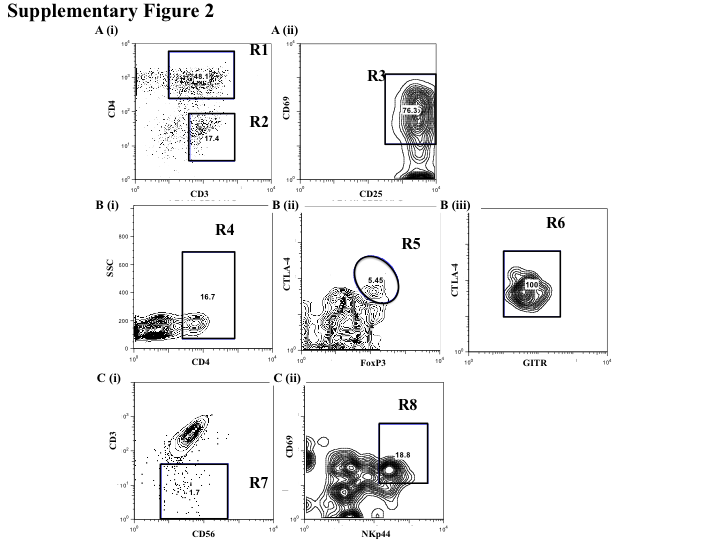

Supplement: Figure S2 — Characterization of activated T cells, Treg cells, and NK cells. A (i) T cells were identified as CD4 T cells and CD8 T cells by CD3+CD4+, R1, and CD3+CD4−, R2, respectively. A (ii) Activated T cells were gated as CD25+CD69+ cells, R3. B (i) CD4+ cells were characterized in R4. B (ii) From R4, the activated Treg cells were gated as FoxP3+CTLA-4+, R5. B (iii) Activated Treg cells were also positive for GITR staining, R6. C (i) NK cells were characterized as CD56+CD3–, R7. C (ii) From R7, activated NK cells were identified as CD69+NKp44+. (TIFF) [file pone.0103254.s002.tiff]

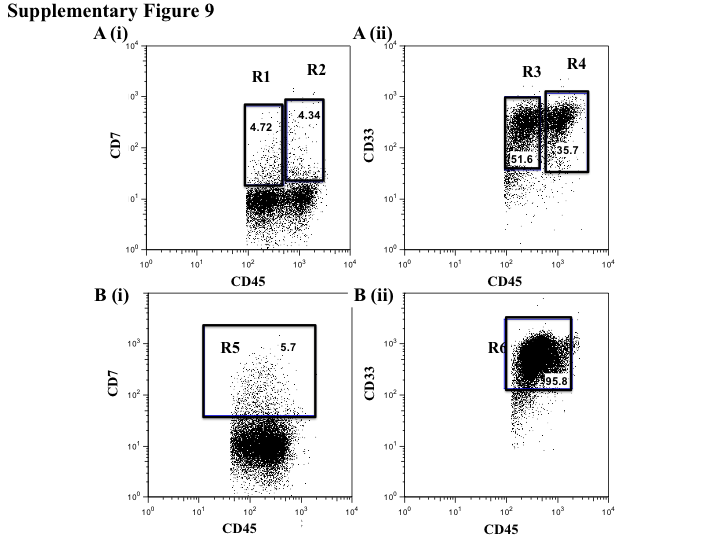

Supplement: Figure S9 — Characterization of lymphoid and myeloid progenitors. A (i) CD45lowCD7+ and CD45highCD7+ CB lymphoid progenitors were gated in R1 and R2 respectively. A (ii) CB myeloid progenitors were identified as CD45lowCD33+ and CD45highCD33+ in R3 and R4 respectively. B (i) PB lymphoid progenitors were gated as CD45+CD7+, R5. B (ii) Myeloid progenitors derived from PB were identified as CD45+CD33+, R6. (TIFF) [file pone.0103254.s009.tiff]
